# 4-1BB Signaling Enhances Primary and Secondary Population Expansion of CD8^+^ T Cells by Maximizing Autocrine IL-2/IL-2 Receptor Signaling

**DOI:** 10.1371/journal.pone.0126765

**Published:** 2015-05-11

**Authors:** Ho S. Oh, Beom K. Choi, Young H. Kim, Don G. Lee, Sunhee Hwang, Myoung J. Lee, Sang H. Park, Yong-Soo Bae, Byoung S. Kwon

**Affiliations:** 1 Cancer Immunology Branch, Division of Cancer Biology, National Cancer Center, Ilsan, Goyang, Gyeonggi, Korea; 2 Immune Cell Production Unit, Program for Immunotherapeutic Research, National Cancer Center, Ilsan, Goyang, Gyeonggi, Korea; 3 Department of Biological Sciences, Sungkyunkwan University, Suwon, Gyeonggi, Korea; 4 Section of Clinical Immunology, Allergy, and Rheumatology, Department of Medicine, Tulane University Health Sciences Center, New Orleans, Louisiana, United States of America; Institut Pasteur, FRANCE

## Abstract

4-1BB (CD137), a member of the tumor necrosis factor receptor superfamily (TNFRSF), is primarily expressed on activated T cells and is known to enhance proliferation of T cells, prevent activation-induced cell death, and promote memory formation of CD8^+^ T cells. In particular, it is well acknowledged that 4-1BB triggering preferentially enhances the expansion of CD8^+^ T cells rather than CD4^+^ T cells, but the underlying mechanism remains unclear. Here we found that 4-1BB triggering markedly increased IL-2Rα (CD25) and IL-2 expressions of CD8+ T cells but minimally for CD4^+^ T cells. Proliferation of CD8^+^ T cells was moderately enhanced by direct 4-1BB triggering in the absence of signaling through IL-2Rα/IL-2 interactions, but further promoted in the presence of IL-2Rα/IL-2 interactions. Among the TNFRSF members including OX40, GITR, CD30, and CD27, 4-1BB was superior in the ability to induce IL-2Rα expression on CD8+ T cells. When the primary and secondary expansions of CD8^+^ T cells *in vivo *were examined by adoptively transferring OVA-specific CD8^+^ T cells along with the treatment with agonistic anti-4-1BB and/or antagonistic anti-CD25 F(ab’)_2_ mAb, 4-1BB triggering enhanced both primary and secondary expansion of CD8^+^ T cells *in vivo*, and the 4-1BB effects were moderately suppressed in primary expansion while completely abolished in secondary expansion of OVA-specific CD8^+^ T cells by blocking IL-2Rα. These results suggest that 4-1BB-mediated increases of IL-2Rα and IL-2 prolong the effects of transient TCR- and 4-1BB-mediated signaling in CD8^+^ T cells, and that 4-1BB triggering preferentially enhances the expansion of CD8^+^ T cells through the amplification of autocrine IL-2/IL-2R signaling loop.

## Introduction

Efficient priming of T cells requires costimulation and cytokine milieu for cell cycle progression, survival and proliferation [1]. 4-1BB (CD137), a member of the tumor necrosis factor receptor superfamily (TNFRSF), is primarily expressed on activated T cells and plays critical roles in preventing activation-induced cell death (AICD), enhancing cytotoxicity of T cells, upregulating survival-related genes, and producing Th1 cytokines such as IL-2, IFN-γ and TNF-α [2, 3]. Although the 4-1BB expression level itself on CD4^+^ T and CD8^+^ T cells is comparable [4], 4-1BB signaling is well-known to preferentially enhance the proliferation of CD8^+^ T cells rather than CD4^+^ T cells *in vitro* and *in vivo* [5, 6]. The underlying mechanisms of such preferential contribution to CD8^+^ T cell proliferation by 4-1BB triggering, however, need to be elucidated.

Antigen-presenting cells (APCs) such as dendritic cells (DCs) uptake Ag at a local area, migrate to adjacent lymph node (LN) for T cell priming, and appear to be matured during their migration along with 4-1BBL expression. Consequently, 4-1BBL-expressing mature DCs are able to efficiently prime T cells, induce 4-1BB on the activated T cells, and transmit 4-1BB signals into T cells by 4-1BBL on mature DCs and possibly activated T cells itself [7]. These 4-1BB/4-1BBL interactions show profound impacts on the proliferation and differentiation of CD8^+^ T cells *in vitro* and *in vivo* [5, 6]. However, since 4-1BB is known to be only transiently expressed on activated T cells at the early stage of proliferation *in vitro* and *in vivo* [2, 8], 4-1BB triggering seems to directly and/or indirectly enhance CD8^+^ T cell responses and 4-1BB effects endure through indirect ways even after 4-1BB expression on activated CD8^+^ T cells decreases.

IL-2 is one of the major positive growth factors for T cells [9, 10]. High levels of IL-2

secreted from CD8^+^ T cell plays important roles in inducing cell-cycle progression [11] and producing cytokines such as IFN-γ [12], and induction of IL-2Rα expression gives rise to memory CD8^+^ T cells [13–15]. 4-1BB triggering enhances IL-2 production from activated T cells [16], and the neutralization of IL-2 inhibits the 4-1BB effects on T cell proliferation *in vitro* [17]. Here we found that 4-1BB triggering markedly increased IL-2Rα expression on activated CD8^+^ T cells rather than CD4^+^ T cells along with an increased IL-2 production. Such 4-1BB-dependent increase of IL-2Rα/IL-2 not only promoted the proliferation of CD8^+^ T cells *in vitro* and *in vivo*, but also was required to increase Ag-specific memory CD8^+^ T cells *in vivo*. These results imply that although 4-1BB expression is an early and transient event in the course of the activation of CD8^+^ T cells, its effects can be boosted and prolonged through the 4-1BB-mediated amplification of IL-2/IL-2Rα signaling in an autocrine manner. The aim of this study was to determine how 4-1BB triggering, after its initial, short-lived expression, displays potent and durable effects on activated CD8^+^ T cells. We hypothesized that such 4-1BB effect may result from a signaling loop that can promote the proliferation of CD8^+^ T cells.

## Materials and Methods

### Mice

C57BL/6 mice were purchased from OrientBio (Gapyeong, Korea). Recombinase-activating gene-2-deficient (Rag2^-/-^), IL-2-deficient (IL-2^-/-^), OT-1 and OT-1 × Thy1.1mice on C57BL/6 background were purchased from the Jackson laboratory (Bar Habor, ME). All mice were maintained under specific pathogen-free conditions in the animal facility of the National Cancer Center in Korea, and were used at 6–8 wk of age. The mice were euthanized by inhalation of carbon dioxide gas using euthanasia chamber. All animal experiments were reviewed and approved by the Animal Care and Use Committee of the National Cancer Center (NCC-10-080) and were performed in accordance with the Guide for the Care and Use of Laboratory Animals.

### Reagents and antibodies

CD4- and CD8- microbeads were purchased from Miltenyi Biotech (Auburn, CA). Anti-CD3 and anti-CD8β mAb for flow cytometry were purchased from BD Pharmingen (San Diego), and PE-Cy5-anti-CD8, FITC- or PE-Cy5-anti-CD4, PE-anti-4-1BB, FITC- or PE-anti-CD25, and FITC- or PE-anti-CD122 were purchased from eBioscience (San Diego, CA). Purified anti-CD16/CD32 (2.4G2), anti-GITR, anti-OX40, anti-CD27, and anti-CD30 mAb were purchased from eBioscience (San Diego, CA). Recombinant human (rh)IL-2 was purchased from PeproTech (Rocky Hill, NJ). LY294002, PD98059, and Triciribine were from Calbiochem (San Diego, CA). Anti-mouse 4-1BB mAb-producing hybridoma cells (3E1) were a gift from Dr. Robert Mittler (Emory University, Atlanta, GA), and anti-CD4 (GK1.5) and anti-CD25 (PC61.5.3) hybridomas were obtained from the American Type Culture Collection (ATCC; Manassas, VA). F(ab’)_2_ fragments of anti-CD25 mAb were generated by 24 h incubation of 10 mg/ml anti-CD25 mAb with immobilized pepsin (Pierce, Rockford, IL). After peptic digestion, the preparation was applied to an immobilized protein G column to remove Fc fragments and any undigested IgG. Purity of the F(ab’)_2_ fragments was checked by SDS-PAGE.

### Isolation and activation of CD4^+^ and CD8^+^ T cells

Lymphocytes were prepared from the spleens and lymph nodes of C57BL/6 and IL-2^-/-^C57BL/6 mice, and then preincubated with Fc blocker 2.4G2 for 10 min at 4 °C. CD4^+^ or CD8^+^ T cells were further isolated by incubating the cells with CD4- or CD8-microbeads. The enriched CD4^+^ or CD8^+^ T cells were >92% pure by flow cytometry, and plated at 3 × 10^5^ cells/well in 96-well round-bottom microplates with 0.1 μg/ml or 0.5 μg/ml of anti-CD3 mAb for 16 h, respectively. The activated T cells were stained with anti-4-1BB-PE along with anti-CD4 or CD8-PE-Cy5, and the percentages of 4-1BB^+^ T cells were routinely ~70% in CD8^+^ T cells and ~50% in CD4^+^ T cells by flow cytometry. For 4-1BB triggering, the anti-CD3-activated CD4^+^ or CD8^+^ T cells were further treated with 5.0 μg/ml of anti-4-1BB mAb or rat IgG after 24 or 48 h.

### CFSE dilution assay

For *in vitro* activation of CD4^+^ or CD8^+^ T cells, T cells were enriched from C57BL/6 mice or OT-1 transgenic mice, and then resuspended in 1 × PBS at 1 × 10^7^ cells/ml and labeled with 10 μM CFSE for 5 min. The CFSE-labeled T cells were quenched with ice-cold FBS for 1 min and washed with complete RPMI medium three times. CFSE-labeled CD4^+^or CD8^+^ T cells were plated at 5 × 10^5^ cells/well in 96-well round-bottom microplates, and stimulated with 0.1 or 0.5 μg/ml of anti-CD3 mAb or 1.0 μg/ml OVA_257-264_ peptide for 16 h, respectively. Then the cells were treated with ant-4-1BB mAb or rat IgG for another 48 h, and were stained with anti-CD8-PE-Cy5 along with anti-CD25 or anti-CD122 mAb. The dilution of CFSE was determined by FACSCalibur (BD Bioscience).

### IL-2 assay

Anti-CD3-activated IL-2^+/+^ or IL-2^-/-^ CD8^+^ T cells for 16 h were treated with rat IgG or anti-4-1BB mAb. Culture supernatants were prepared at 0, 1, 2, 4, 6, 12, 24, 48, and 72 h after 4-1BB triggering, and IL-2 concentrations were measured using BD Cytometric Bead Array (CBA) Mouse IL-2 Flex Set (BD Bioscience) on a FACSCalibur cytometer equipped with CellQuestPro and CBA software.

### Serum cytokines

Seven days after Thy1.1^+^ OT-1 transferred C57BL/6 mice had been challenged with 20 μg of whole OVA protein-incomplete Freund's adjuvant (IFA) emulsion and 100 μg of agonistic anti-4-1BB mAb or rat IgG from day 0. Some of the mice were injected i.p. with 100 μg of anti-CD25 F(ab’)_2_ every 5 days two times from day 0. Then the serum was collected from each mouse. Serum cytokines were quantified using a cytometric bead array kit (BD Biosciences) on a FACSCalibur cytometer equipped with CellQuestPro and CBA software.

### [^3^H]-thymidine incorporation assay

CD8^+^ T cells were enriched by MACS magnetic separation system from IL-2^+/+^ and IL-2^-/-^ C57BL/6 mice, then the cells were plated in 96-well round-bottom plates at a concentration of 2–3 × 10^5^ cells/well, and stimulated with 0.1 μg/ml of anti-CD3 mAb for 16 h. The activated CD8^+^ T cells were preincubated with the indicated dose of anti-CD25 mAb for 1 h and further treated with rat IgG, anti-4-1BB mAb for another 48 h. The cells were labeled with 1.0 μCi of [^3^H]-thydimine for the last 8 h to assess the proliferation, and the incorporation of thymidine was measured by liquid scintillation spectroscopy.

### Adoptive CD8^+^ T cell transfer *in vivo*

CD8^+^ T cells were enriched from Thy1.1^+^ OT-1 transgenic mice using CD8-microbeads, and transferred into Thy1.2^+^ C57BL/6 mice at a density of 2 × 10^6^ cells/mouse via intravenous (i.v.) route at day 0. Whole OVA protein-incomplete IFA emulsion was subcutaneously (s.c.) injected into the flanks of the mice at a dose of 20 μg per mice on day 0 and administered intraperitoneally (i.p.) with 100 μg of agonistic anti-4-1BB mAb or rat IgG every 7 days three times from day 0. Some of the mice were injected i.p. with 100 μg of anti-CD25 F(ab’)_2_ every 5 days four times from day 0. For the secondary expansion of OT-1 CD8^+^ T cells, whole OVA-IFA emulsion (20 μg per mice) was injected into the flanks of the mice at day 21. The frequencies of Thy1.1^+^ CD8^+^ T cells were analyzed in draining inguinal lymph nodes (LNs) on days 0, 7, 14, 21, and 26 by flow cytometry, and the absolute numbers of Thy1.1^+^ CD8^+^ T cells were calculated by multiplying the total number of viable cells by the calculated percentages.

### Statistical analysis

All data were analyzed with the statistical program, Prism4.0 GraphPad (San Diego, CA). Student’s *t*-test was used to determine the statistical significance of differences between groups.

## Results

### 4-1BB triggering increases IL-2Rα/β expressions on activated CD4^+^ and CD8^+^ T cells

**Fig 1:**
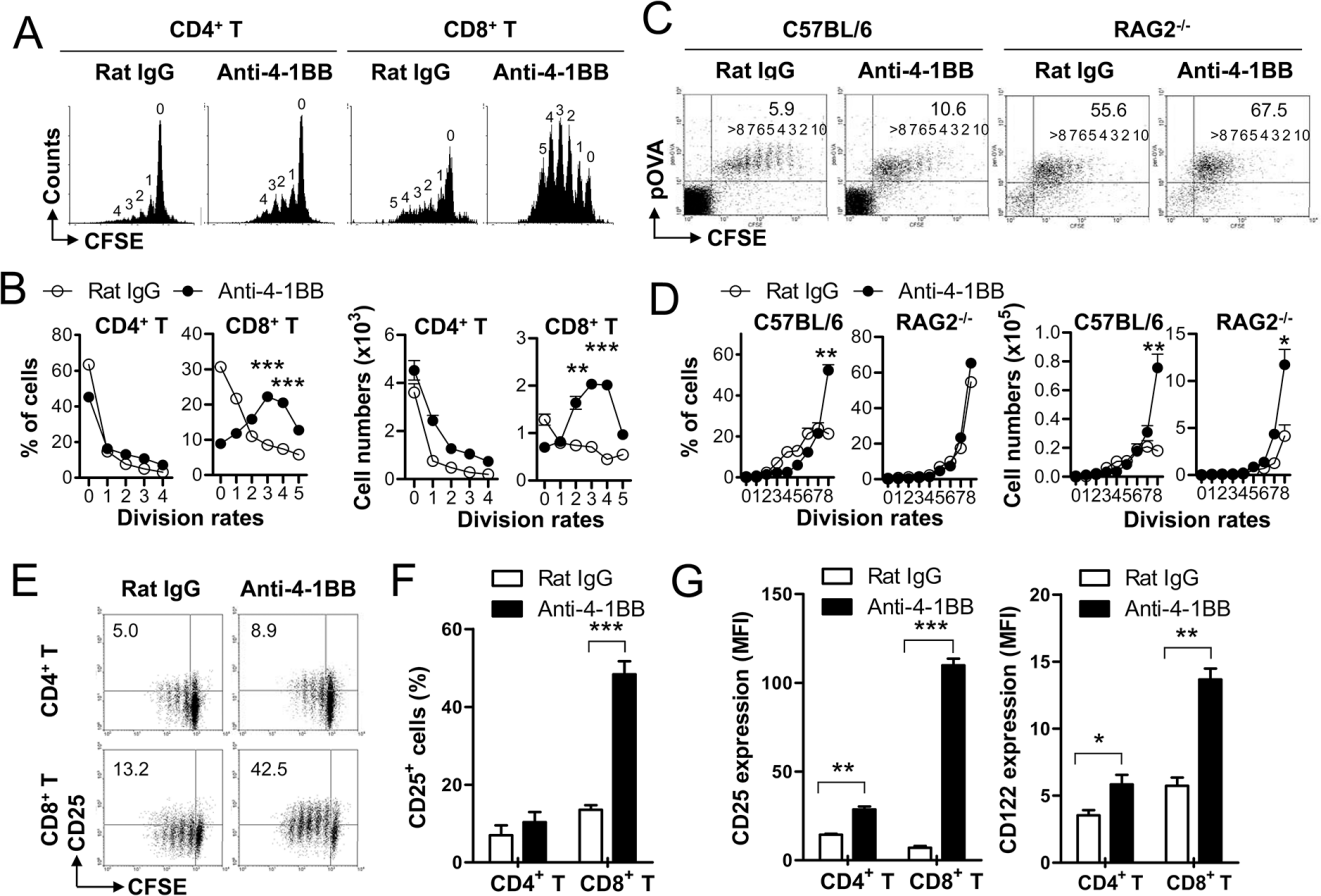
IL-2Rα and IL-2Rβ expressions on CD4^+^ and CD8^+^ T cells following 4-1BB triggering. (A) CD4^**+**^ T and CD8^**+**^ T cells isolated from C57BL/6 mice were labeled with CFSE, activated with 0.1 μg/ml of anti-CD3 mAb for 16 h, and further treated with anti-rat IgG or 5 μg/ml of anti-4-1BB mAb for another 48 h. The cells were stained with the indicated antibodies and subsequently analyzed by FACSCalibur (BD Bioscience). CFSE dilution of CD4^**+**^ T and CD8^**+**^ T cells 48 h after the treatment of anti-rat IgG or anti-4-1BB mAb. Values represent the mean number of divisions (A). Percentages of each viable dividing cells were calculated using BD CellQuest software. The absolute numbers of dividing T cells were calculated by multiplying the total number of viable cells by the calculated percentages (B). (C-D) C57BL/6 or RAG2^**-/-**^mice were injected i.v. with CFSE-labeled OT-1 CD8^**+**^ T cells (2 × 10^**6**^ cells/mice), immunized with the emulsified OVA into the flank sites via s.c. route, and further injected i.p. with anti-rat IgG or anti-4-1BB mAb at days 0 and 2. Inguinal LN cells were prepared from each group of mice at day 5, stained with anti-CD8 and H-2K^**b**^/OVA257-564 pentamer, and subsequently analyzed by FACSCalibur (BD Bioscience) (C). Percentages and absolute numbers of each dividing T cells were assessed as described above (D). (E-G) CD4^**+**^ T and CD8^**+**^ T cells were cultured as described in (A), CD4- or CD8-gated cells were plotted as CFSE vs. CD25 (E). Anti-rat IgG- or anti-4-1BB-treated CD4^**+**^ or CD8^**+**^ T cells for 48 h were stained with anti-CD25 (IL-2Rα) or anti-CD122 (IL-2Rβ) along with anti-CD4 or anti-CD8 mAb. Percentages of CD25-expressing CD4^**+**^ or CD8^**+**^ T cells were calculated (F). MFIs of CD25 and CD122 were calculated using BD Cell Quest software (BD Bioscience) (G). Data are representative of at least three independent experiments. Results in B, D, F, and G are mean ± SD (n = 3; *, p< 0.05; **, p< 0.01;***, p< 0.001). MFI: mean fluorescence intensities.

To determine whether 4-1BB triggering enhances expansion of CD4^+^ T and CD8^+^ T cells by promoting cell division or by preventing apoptosis, CFSE-labeled CD4^+^ T and CD8^+^ T cells were activated with anti-CD3 mAb for 16 h to induce 4-1BB on their surface, and further treated with rat IgG or agonistic anti-4-1BB mAb for another 48 h. CFSE dilutions indicated that CD4^+^ T cells divided 0–4 times and CD8^+^ T cells divided 0–5 times following the treatment with anti-CD3 mAb, which appeared to be minimally enhanced by treating with anti-4-1BB mAb (Fig 1A). However, the same anti-4-1BB mAb treatment markedly increased the frequencies of each dividing T cells (Fig 1A). Statistical analysis indicated that anti-4-1BB mAb treatment significantly increased the frequencies and numbers of each dividing T cells—particularly for CD8^+^ T cells than CD4^+^ T cells (Fig 1B).

To examine whether these *in vitro* findings also occurred *in vivo*, CFSE-labeled OT-1 CD8^+^ T cells were adoptively transferred into C57BL/6 or Rag2^-/-^ mice, immunized with OVA, and further injected with rat IgG or anti-4-1BB mAb. CFSE dilutions of OT-1 CD8^+^ T cells were analyzed in inguinal LNs of C57BL/6 mice on day 5. Frequencies of OT-1 CD8^+^ T cells were increased by ~2 fold following the treatment with anti-4-1BB mAb and 4-1BB triggering resulted in the massive accumulation of the OT-1 CD8^+^ T cells that had divided > 8 times, while in rat IgG-treated mice, the OT-1 CD8^+^ T cells that had divided 4–8 times were dominant populations (Fig 1C; left panels). In RAG2^-/-^ mice, OT-1 CD8^+^ T cells divided > 8 times independent of 4-1BB triggering, but the frequencies of OT-1 CD8^+^ T cells were increased by treating with anti-4-1BB (Fig 1C; right panels). Since lymphocyte-deficient RAG2^-/-^ mice provoke the lymphopenia-driven proliferation of T cells *in vivo* [18–20], these data indicate that 4-1BB triggering may generate the environment that is similar to the condition for lymphopenia-driven proliferation of T cells. Again, statistical analysis indicated that the treatment with anti-4-1BB significantly increased the frequency of CD8^+^ T cells that divided > 8 times only in C57BL/6 (Fig 1D; left panel), while some treatment significantly increases the absolute numbers of CD8^+^ T cells that divided > 8 times in both C57BL/6 and RAG2^-/-^ mice (Fig 1D; right panel). These results indicate that 4-1BB triggering enhances the expansion of CD8^+^ T cells *in vitro* and *in vivo* by accumulating the dividing CD8^+^ T cells possibly through the prevention of AICD.

In our previously performed microarray using CD8^+^ T cells that were isolated from HSV-1-infected C57BL/6 mice following rat IgG or anti-4-1BB mAb treatment *in vivo* (unpublished data), we found that 4-1BB triggering increased various types of membrane proteins including KLRG1, CCR2, CCR5, TIM-3, IL-2Rβ, and galectin-3. Since IL-2 neutralization has been reported to decrease the 4-1BB-mediated enhancement of CD8^+^ T cell proliferation [17], we examined whether 4-1BB triggering would increase IL-2Rα/β expressions on the activated CD4^+^ and CD8^+^ T cells. Anti-CD3-activated CD4^+^ and CD8^+^ T cells for 16 h were stimulated with rat IgG or anti-4-1BB mAb for 48 h, and IL-2Rα expression was analyzed by flow cytometry. 4-1BB triggering increased IL-2Rα expression on both of the dividing CD4^+^ T and CD8^+^ T cells—especially for CD8^+^ T cells than CD4^+^ T cells (Fig 1E). 4-1BB triggering significantly increased the percentages of IL-2Rα-expressing CD4^+^ or CD8^+^ T cells (Fig 1F). The mean fluorescence intensity (MFI) values for IL-2Rα expression indicated that 4-1BB triggering increased IL-2Rα expression by ~2 fold in CD4^+^ T cells, while by ~15 fold in CD8^+^ T cells (Fig 1G; left panel). In the case of IL-2Rβ (CD122), 4-1BB triggering increased the MFI of IL-2Rβ by ~1.3 fold in CD4^+^ T cells and by ~2 fold in CD8^+^ T cells (Fig 1G; right panel). These results suggest that 4-1BB triggering not only preferentially enhances the expansion of CD8^+^ T cells, but also preferentially increases the levels of IL-2R on CD8^+^ T cells.

### 4-1BB triggering amplifies autocrine IL-2/IL-2Rα signaling loop in CD8^+^ T cells

**Fig 2:**
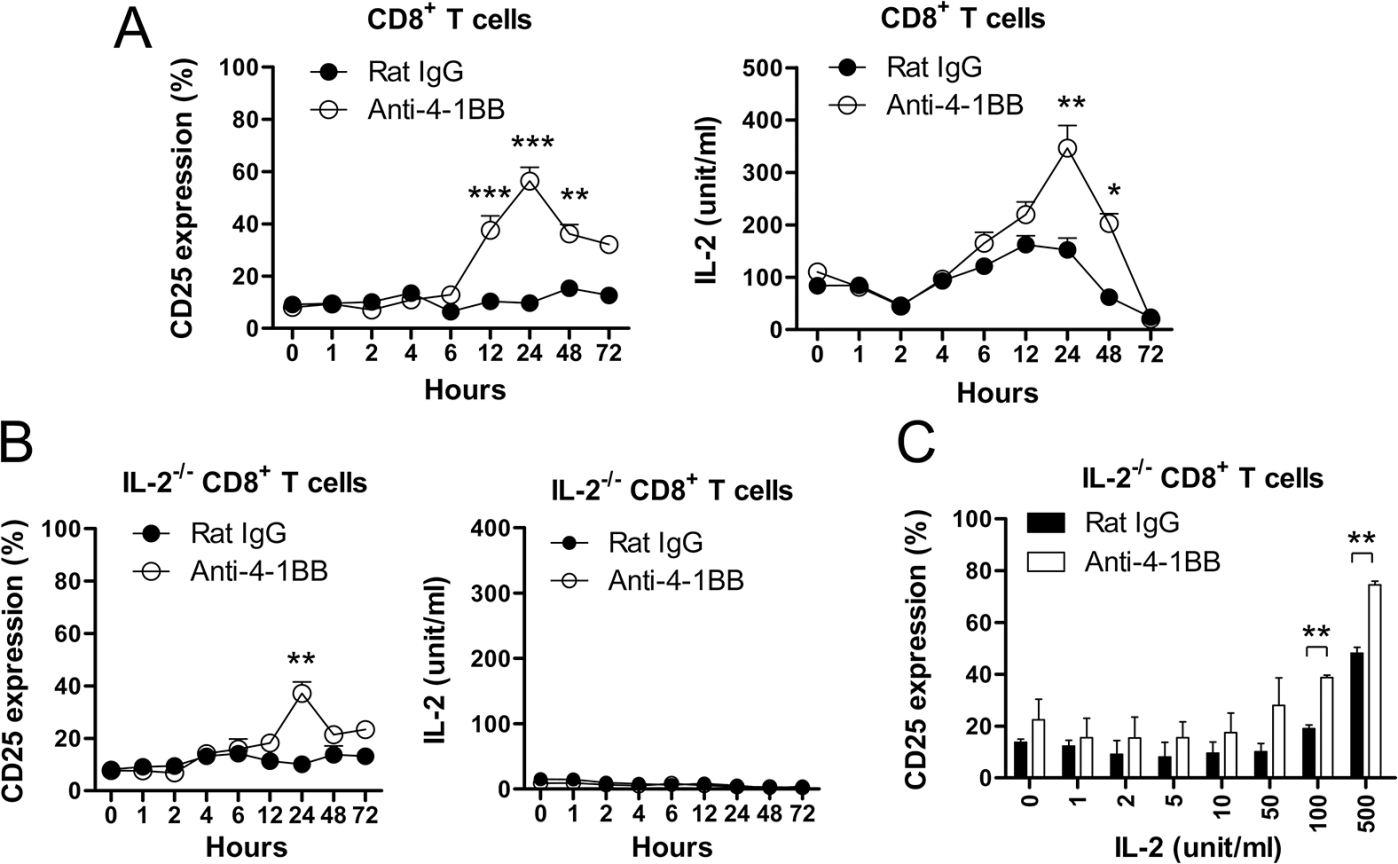
Induction of IL-2Rα on CD8^+^ T cells by 4-1BB triggering and 4-1BB-mediated IL-2 production. (A) Anti-CD3-activated CD8^**+**^ T cells for 16 h were further stimulated with anti-rat IgG or anti-4-1BB mAb for the indicated time points. The CD8^**+**^ T cells were stained with anti-CD8 and anti-CD25 mAb, and subsequently analyzed by FACSCalibur (BD Bioscience). IL-2 production was assessed using BD Cytometric Bead Array Mouse IL-2 Flex Set (BD Bioscience). (B) CD8^**+**^ T cells from IL-2^**-/-**^ mice were activated with anti-CD3 mAb for 16 h and further stimulated with anti-rat IgG or anti-4-1BB mAb for the indicated time points. CD25 expression on CD8^**+**^ T cells and IL-2 production were assessed as described above. (C) Anti-CD3-activated IL-2^**-/-**^ CD8^**+**^ T cells for 16 h were treated with anti-rat IgG or anti-4-1BB mAb for another 48 h in the presence of the indicated dose of IL-2. CD25 expression on CD8^**+**^ T cells was analyzed by flow cytometry. Data are representative of three independent experiments. Results are mean ± SD (n = 5; *, p< 0.05; **, p< 0.01;***, p< 0.001).

We next examined the kinetics of IL-2Rα expression and IL-2 production of CD8^+^ T cells after 4-1BB triggering. Anti-CD3-activated CD8^+^ T cells were treated with the rat IgG or anti-4-1BB mAb for 48 h, and IL-2Rα expression was assessed by flow cytometry. IL-2Rα was significantly increased on CD8^+^ T cells 12 h after 4-1BB triggering, which culminated at 24 h, and then gradually declined (Fig 2A; left panel). The production kinetics of IL-2 was similar to the IL-2Rα expression kinetics by showing that the IL-2 production peaked at 24 h after 4-1BB triggering (Fig 2A; right panel). The decline of IL-2Rα and IL-2 levels 24 h after 4-1BB triggering indicated that the increased IL-2 might be consumed by the increased IL-2Rα.

Next we wondered whether the 4-1BB-meidated increase of IL-2 contributed to inducing IL-2Rα expression on CD8^+^ T cells. CD8^+^ T cells from IL-2^-/-^ mice were isolated and activated with anti-CD3 mAb for 16 h and further stimulated with rat IgG or anti-4-1BB mAb for the indicated time points. 4-1BB triggering significantly increased IL-2Rα expression on IL-2^-/-^ CD8^+^ T cells although there was no production of IL-2 (Fig 2B). However, the IL-2Rα expression on IL-2^-/-^ CD8^+^ T cells was lower than that of IL-2-intact CD8^+^ T cells. When the anti-CD3-activated IL-2^-/-^ CD8^+^ T cells were treated with the indicated dose of exogenous IL-2 in the presence of rat IgG or anti-4-1BB mAb for 48 h, IL-2Rα expression was significantly increased by 4-1BB triggering under > 100 IU/ml IL-2 conditions (Fig 2C).

These results indicate that 4-1BB triggering augments IL-2Rα expression and IL-2 production of CD8^+^ T cells with delayed kinetics, and the increased IL-2, in turn, heightens and sustains IL-2Rα expression on CD8^+^ T cells. This suggests that 4-1BB triggering enhances IL-2 and IL-2Rα expression of CD8^+^ T cells in both IL-2-independent and-dependent manners.

### 4-1BB triggering increases IL-2 expression to enhance the CD8^+^ T cell proliferation

**Fig 3:**
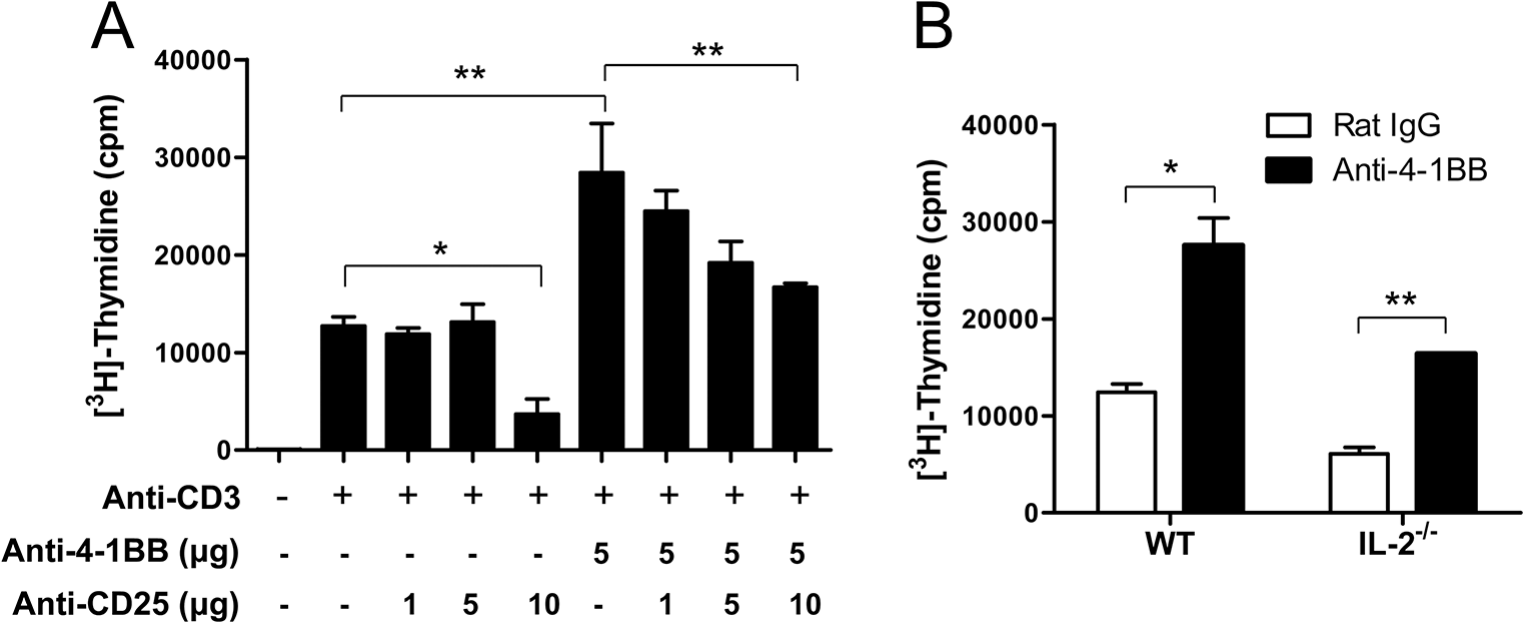
Blockade of IL-2/IL-2R signaling reduces 4-1BB-mediated enhancement of CD8^+^ T cell proliferation. (A) Anti-CD3-activated CD8^**+**^ T cells for 16 h were preincubated with the indicated dose of anti-CD25 mAb and further treated with anti-rat IgG or anti-4-1BB mAb for another 48 h. (B) Anti-CD3-activated IL-2^**-/-**^ CD8^**+**^ T cells for 16 h were treated with anti-rat IgG or anti-4-1BB mAb for another 48 h, and labeled with [^**3**^H]-thydimine for the last 8 h. Incorporation of thymidine was measured using a beta scintillation counter. The results are represented as means ± SD of triplicates. Results are mean ± SD (n = 5; *, p< 0.05; **, p< 0.01;***, p< 0.001).

Since we found that 4-1BB triggering markedly increased IL-2 and IL-2Rα on CD8^+^ T cells (Fig 2A), we next examined whether the increased IL-2 and IL-2Rα would be crucial for 4-1BB-mediated promotion of CD8^+^ T cell expansion. Anti-CD3-activated CD8^+^ T cells for 16 h were preincubated with the indicated dose of anti-CD25 mAb for 1 h, and further treated with rat IgG or anti-4-1BB mAb for 48 h. Thymidine incorporation indicated that 4-1BB triggering enhanced CD8^+^ T cell expansion by ~2.2 fold and the blockade of IL-2Rα signaling significantly diminished CD8^+^ T cell expansion in a dose-dependent manner (Fig 3A). However, the expansion rate was still higher in 4-1BB-triggered CD8^+^ T cells compared with that of rat IgG-treated CD8^+^ T cells even in the absence of IL-2R signaling (Fig 3A). Similar to these results, when anti-CD3-activated IL-2^-/-^ CD8^+^ T cells were treated with rat IgG or anti-4-1BB mAb for 48 h, the expansion rates were attenuated in IL-2^-/-^ CD8^+^ T cells compared with that of normal CD8^+^ T cells, but 4-1BB triggering still enhanced CD8^+^ T cell expansion in the absence of IL-2 (Fig 3B).

These results indicate that 4-1BB signaling enhances the expansion of CD8^+^ T cells in both IL-2-independent and-dependent manners, and that the increased IL-2 boosts the expansion of activated CD8^+^ T cells.

### 4-1BB signaling increases IL-2Rα expression through PI3K-ERK signals and is superior to other TNFRSF members in inducing IL-2Rα expression on CD8^+^ T cells

**Fig 4:**
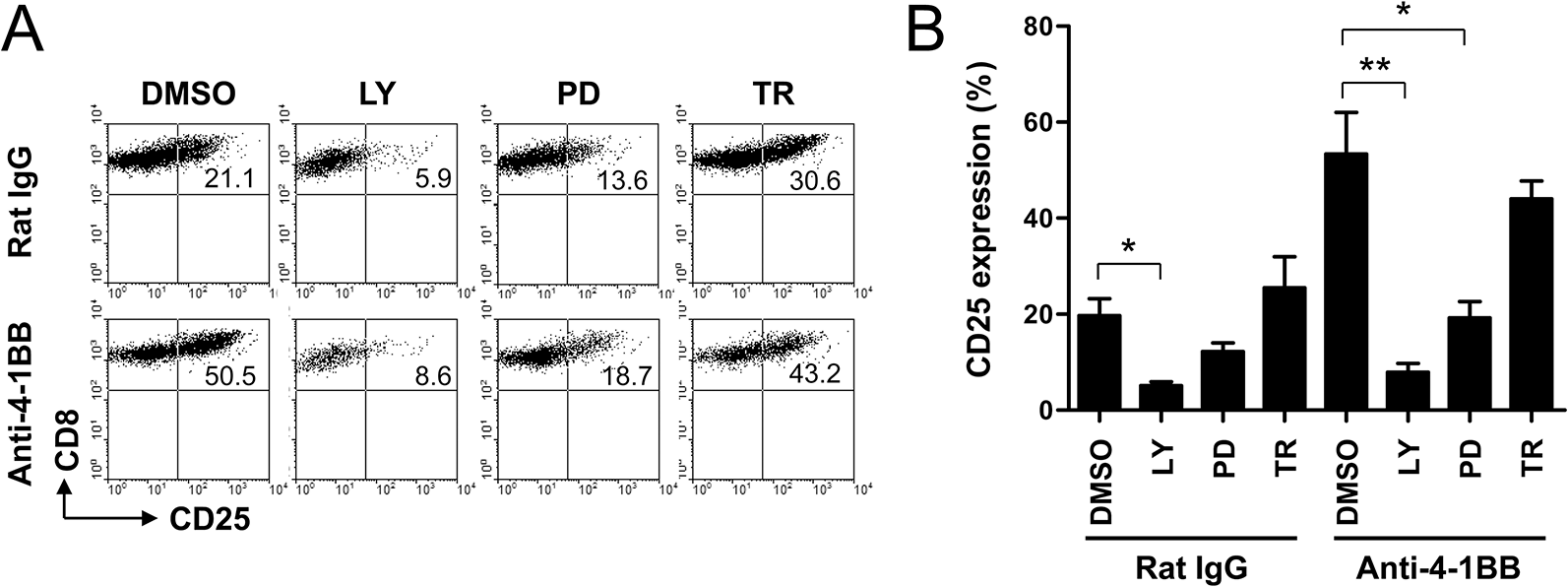
4-1BB-mediated CD25 induction on CD8^+^ T cells in the presence of PI3K, ERK, or AKT inhibitor. (A-B) Anti-CD3-activated CD8^**+**^ T cells for 16 h were pre-incubated with 20 μM LY294002 (LY; PI3K inhibitor), 30 μM PD98059 (PD; ERK inhibitor), or 2 μM Triciribine (TR; AKT inhibitor) for 1 h and then treated with anti-rat IgG or anti-4-1BB mAb for another 24 h. The CD8^**+**^ T cells were stained with anti-CD8 and anti-CD25 mAb, and subsequently analyzed by FACSCalibur (BD Bioscience). Live CD8^**+**^ T cells were gated and plotted CD8 vs. CD25 (A). Percentages of CD25^**+**^ CD8^**+**^ T cells were calculated and represented as mean ± SD (n = 5; *, p< 0.05; **, p< 0.01) (B).

4-1BB triggering is known to directly activate the PI3K-ERK pathway and sustain AKT signals with the delayed kinetics [4]. Therefore, we next examined the roles of PI3K, ERK and AKT in the induction of IL-2Rα expression on CD8^+^ T cells. Anti-CD3-activated CD8^+^ T cells were treated with rat IgG or anti-4-1BB mAb in the presence of inhibitors specific for PI3K, ERK, and AKT. IL-2Rα expressions were completely decreased by treating with rat IgG- or anti-4-1BB mAb-treated CD8^+^ T cells with PI3K inhibitor, partially by ERK inhibitor, but not by AKT inhibitor (Fig 4A). Statistical analysis also demonstrated that the blockade of PI3K and ERK significantly reduced IL-2Rα expression on both rat IgG- or anti-4-1BB mAb-treated CD8^+^ T cells (Fig 4B). Given that PI3K functions as a key molecule for the TCR-mediated mitogenic signaling pathway [21], these results indicate that 4-1BB triggering amplifies TCR-mediated mitogenic signals via PI3K and thus, induces IL-2Rα expression on the activated CD8^+^ T cells.

**Fig 5:**
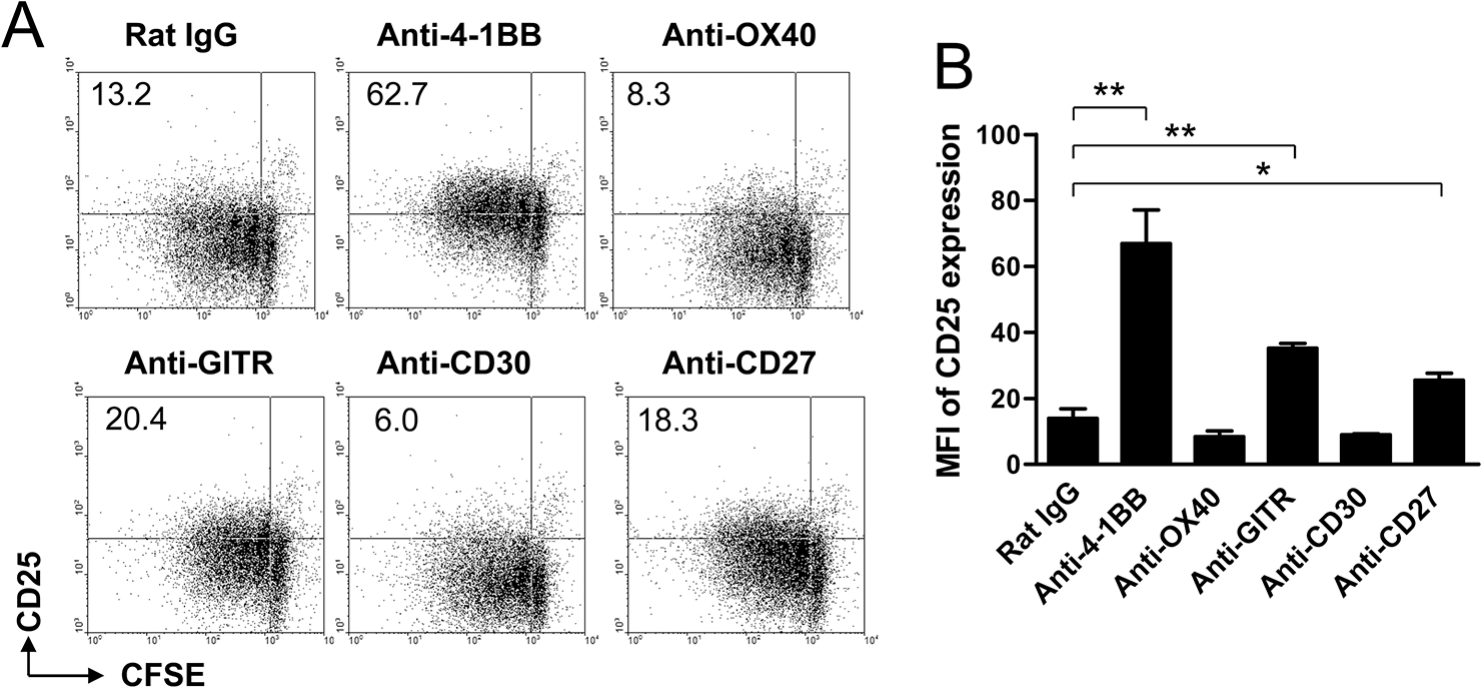
IL-2Rα induction on CD8^+^ T cells by TNFRSF members. (A-B) CFSE-labeled CD8^**+**^ T cells were activated with 0.1 μg/ml of anti-CD3 mAb and simultaneously treated with 5 μg/ml of anti-rat IgG, anti-4-1BB, anti-OX40, anti-GITR, anti-CD30, or anti-CD27 mAb for 3 days. The cells were stained with anti-CD8 and anti-CD25 mAb. CD8-gated cells were plotted as CFSE vs. CD25 (A) and MFIs of CD25 expression were calculated using BD CellQuest software (BD Bioscience) (B). Data are representative of at least three independent experiments. Result in B is mean ± SD (n = 3; *, p< 0.05; **, p< 0.01).

Since 4-1BB triggering preferentially enhances CD8^+^ T cell proliferation [5, 6], we wondered whether 4-1BB was superior to other TNFRSF members in inducing IL-2Rα expression on CD8^+^ T cells. CFSE-labeled CD8^+^ T cells were activated with anti-CD3 mAb and cultured in the presence of agonistic forms of mAb specific for 4-1BB, OX40, GITR, CD30, and CD27 for 3 days. Again, 4-1BB triggering markedly increased IL-2Rα expression on the activated CD8^+^ T cells, while signaling through other TNFRSF members marginally or moderately increased IL-2Rα expression on CD8^+^ T cells (Fig 5A). Statistical analysis demonstrated that the signaling through 4-1BB, GITR, and CD27 significantly increased IL-2Rα expression on CD8^+^ T cells, but the IL-2Rα induction rate was higher in 4-1BB-stimulated CD8^+^ T cells compared to that of GITR- or CD27-triggered CD8^+^ T cells (Fig 5B).

### 4-1BB-mediated increase of IL-2 boosts primary and secondary CD8^+^ T cell responses *in vivo*

**Fig 6:**
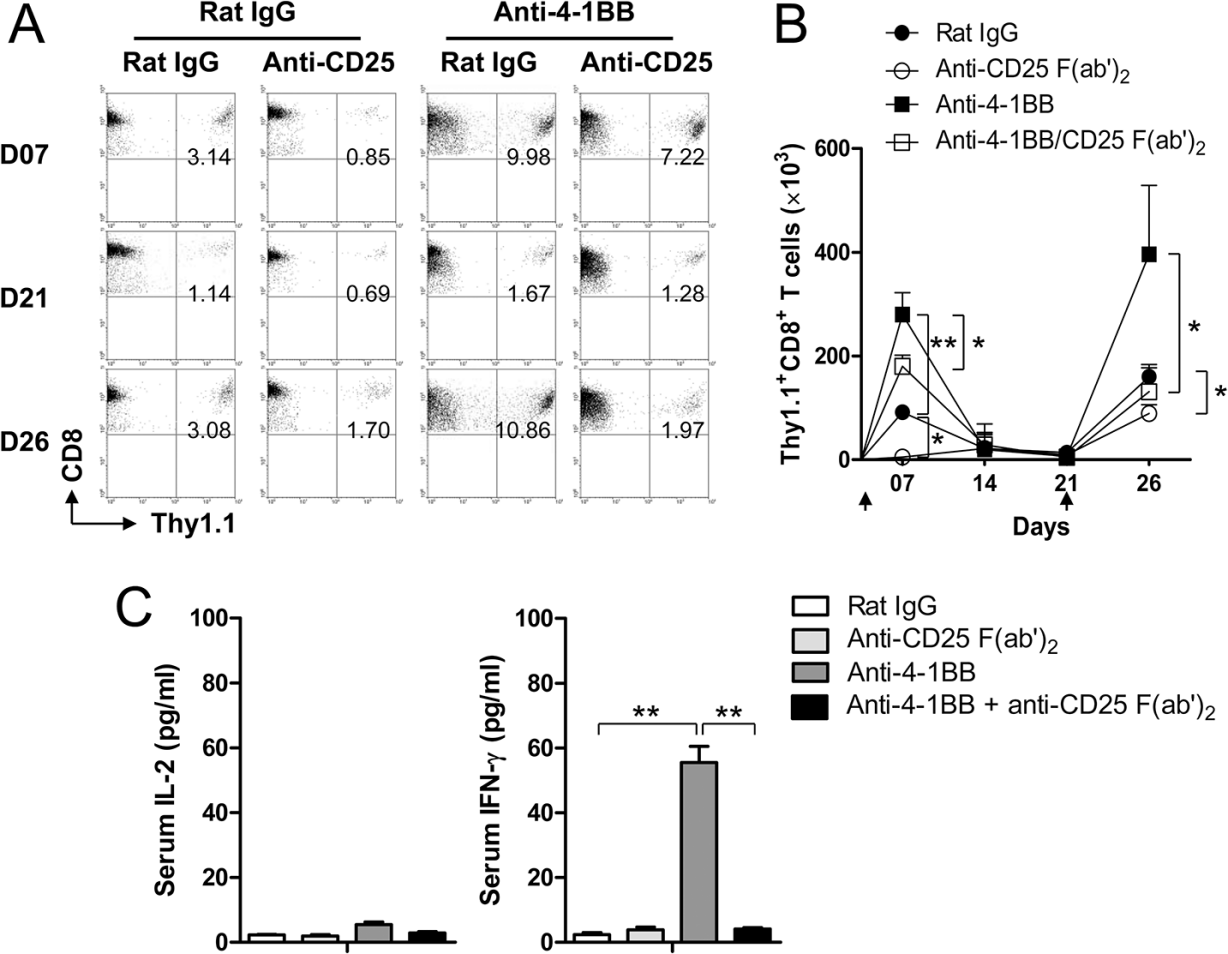
4-1BB-mediated memory formation of CD8^+^ T cells in the absence of IL-2/IL-2R signaling. (A-C) C57BL/6 mice were injected i.v. with Thy1.1^**+**^ OT-1 CD8^**+**^ T cells (2 × 10^**6**^ cells/mice) and immunized s.c. with 20 μg of whole OVA-emulsified in IFA. Anti-rat IgG or anti-4-1BB mAb were administered to the immunized mice at day 0, 7, and 14, and anti-CD25 F(ab’)_2_ was given at days 0, 5, 10, and 15 via i.p. route. The mice were re-challenged with 20 μg of whole OVA-emulsified in IFA at days 21 to boost memory T cells. Inguinal LN cells were prepared from each group of mice at 7, 14, 21 or 26 days, stained with anti-CD8 and anti-Thy1.1 mAb, and subsequently analyzed by FACSCalibur (A). Absolute numbers of Thy1.1^**+**^ CD8^**+**^ T cells in inguinal LNs at the indicated days (B). Serum IL-2 and IFN-γ were assessed using BD Cytometric Bead Array Mouse IL-2 & IFN-γ Flex Set (BD Bioscience) (C). Arrows indicate the days of immunization. Results in B and C are mean ± SD (n = 3; *, p< 0.05; **, p< 0.01). LN: lymph node.

Since IL-2 is essential for the expansion and memory cell formation of CD8^+^ T cells [22, 23], we examined whether the increased IL-2 would be required to enhance the CD8^+^ T cell responses by 4-1BB triggering. OVA-specific CD8^+^ T cells from Thy1.1^+^ OT-1 mice were adoptively transferred into Thy1.2^+^ C57BL/6 mice, immunized with whole OVA, and further treated with rat IgG or anti-4-1BB mAb along with or without antagonistic anti-CD25 F(ab’)_2_. The primary CD8^+^ T cell responses were analyzed 7 days after the OVA immunization and the secondary CD8^+^ T cell responses were 5 days after the re-challenge of OVA at day 21. When the draining inguinal LN cells were analyzed on day 7, the frequencies of the transferred Thy1.1^+^ CD8^+^ T cells were increased by treating the mice with anti-4-1BB mAb and decreased by anti-CD25 F(ab’)_2_ treatment (Fig 6A). The increased frequency of Thy1.1^+^ CD8^+^ T cells by 4-1BB triggering was moderately diminished by anti-CD25 F(ab’)_2_ treatment (Fig 6A). On day 21, each group of mice was re-challenged with OVA for 5 days and the frequencies of Thy1.1^+^ CD8^+^ T cells were assessed by flow cytometry. In the secondary response, OVA-specific CD8^+^ T cells were markedly increased in the anti-4-1BB mAb-treated mice but this pattern was reversed by treating with anti-CD25 F(ab’)_2_ mAb (Fig 6A).

Absolute numbers of Thy1.1^+^ CD8^+^ T cells in the draining LNs demonstrated that OVA-specific CD8^+^ T cells were significantly increased by treating with anti-4-1BB mAb in both primary and secondary CD8^+^ T cell expansion, whereas significantly decreased by treating with anti-CD25 F(ab’)_2_ mAb (Fig 6B). Notably, anti-CD25 F(ab’)_2_ treatment was more effective in reducing the enhanced CD8^+^ T cell responses by 4-1BB triggering in the secondary T cell expansions rather than the primary ones (Fig 6B). When IL-2 and IFN-γ levels were assessed in serum 7 days after OT-1 CD8^+^ T cell transfer, we found that IL-2 was minimally detected in the serum in any condition while IFN-γ was significantly increased by 4-1BB triggering but not in the presence of anti-CD25 F(ab’)_2_ mAb (Fig 6C).

Taken together, these results indicate that 4-1BB triggering enhanced the expansion of CD8^+^ T cells through IL-2-dependent and—independent manners during the effector phase. 4-1BB-mediated enhancement of IL-2 production was crucial in generating memory CD8^+^ T cells and IFN- γ-producing effector CD8^+^ T cells.

## Discussion

Here we demonstrate that *in vitro* 4-1BB triggering minimally enhances the division rate of CD4^+^ T and CD8^+^ T cells, but markedly increases the accumulation of live dividing CD8^+^ T cells (Fig 1A). 4-1BB triggering preferentially increased IL-2 and IL-2Rα expression of CD8^+^ T cells, and the increased IL-2/IL-2Rα expression, in turn, stimulated CD8^+^ T cell expansion. In addition, the increased IL-2/IL-2Rα expression by 4-1BB triggering not only contributed to enhancing the primary CD8^+^ T cell expansion but also increasing memory CD8^+^ T cells *in vivo* (Fig 6). These results suggest that 4-1BB triggering preferentially enhances CD8^+^ T cell expansion by selectively amplifying the IL-2/IL-2R signaling pathway.

It is well established that 4-1BB triggering enhances the proliferation of CD8^+^ T cells *in vitro* and *in vivo* [5, 6]. In this study, CFSE dilution assay rather than [^3^H]-thymidine incorporation assay was conducted to identify whether 4-1BB triggering would enhance CD8^+^ T cell expansion by preventing AICD or by promoting cellular division. The results indicate that 4-1BB triggering primarily enhances the CD8^+^ T cell expansion by rescuing the dividing CD8^+^ T cells from apoptosis, but minimally by promoting cell cycle progression *in vitro* and *in vivo* (Fig 1A and 1D). The most abundant division rates of the transferred OT-1 CD8^+^ T cells were 4–8 times in rat IgG-treated C57BL/6 mice and > 8 times in anti-4-1BB-treated C57BL/6 mice. In lymphopenic RAG2^-/-^ mice, however, the majority of the transferred CD8^+^ T cells were divided > 8 times independent of 4-1BB triggering (Fig 1C and 1D). Naïve CD8^+^ T cells undergo proliferation without antigenic stimulus under lymphopenic condition [18–20]. Since the effects of 4-1BB triggering on CD8^+^ T cell divisions in C57BL/6 mice were comparable with those in RAG2^-/-^ mice (Fig 1C), these data imply that 4-1BB triggering promotes the CD8^+^ T cell proliferation by generating cytokine milieu such as IL-7 and IL-15 that may provide a favorable condition for T cell expansion [24].

IL-2/IL-2R interactions have been reported to have crucial roles in modulating CD8^+^ T cell survival and differentiation [9, 25]. Indeed, T cell responses are impaired in IL-2-deficient mice [22] and 4-1BB triggering requires IL-2 to enhance T cell responses *in vitro* [17]. 4-1BB triggering only transiently increased IL-2Rα expression on the IL-2-deficient CD8^+^ T cells (Fig 2B), while the IL-2Rα expression was enhanced and prolonged in the IL-2-competent CD8^+^ T cells by 4-1BB triggering. This indicates that IL-2/IL-2R interactions *per se* are involved in the IL-2Rα expression (Fig 2A). 4-1BB triggering increased the IL-2Rα expression in IL-2-dependent and-independent manners, and the increased IL-2 was required to enhance the primary expansion of CD8^+^ T cells in part and to generate memory CD8^+^ T cells *in vivo* (Fig 6). Given that Ag-pulsed and 4-1BBL-expressing DCs would activate naïve CD8^+^ T cells and induce 4-1BB on CD8^+^ T cells along with their activation in the early phase of immune activation, 4-1BB signaling may be functional only in the initial phase of CD8^+^ T cell expansion. Although CD8^+^ T cells exposed to a foreign Ag transiently induce 4-1BB expression on their surface [2, 8], 4-1BB signaling appears to continuously tune the fate of CD8^+^ T cells through the increase of IL-2/IL-2R expressions even after the decline of 4-1BB expression.

4-1BB is a unique molecule among TNFRSF members in that it preferentially enhances CD8^+^ T cell responses rather than those of CD4^+^ T cells [5, 6]. Here we found that only 4-1BB triggering among the TNFRSF members including OX40, GITR, CD30 and CD27 markedly induced IL-2 and IL-2Rα expressions in CD8^+^ T cells but not in CD4^+^ T cells (Fig 5). IL-2 is known to be produced primarily by the activated CD4^+^ T cells and thus helps the expansion and memory formation of activated CD8^+^ T cells in a paracrine way [9–12]. A study using a WT and IL-2R-deficient bone marrow chimera demonstrated that IL-2 is crucial during the primary immune responses in programming the development of memory CD8^+^ T cells [26]. However, a recent study noted that CD8^+^ T cells, rather than DCs or CD4^+^ T cells, need to produce IL-2 to generate memory CD8^+^ T cells and CD4^+^ T cells provide the help for the memory formation by activating DCs through CD40L-CD40 interactions [22]. Indeed, expansion of IL-2^-/-^ OT-1 CD8^+^ T cells during infection is comparable with that of wild-type OT-1 cells in the primary response but show profound defect in the secondary response [22]. In the absence of IL-2/IL-2R signaling, 4-1BB triggering enhanced the primary expansion of transferred CD8^+^ T cells but not the secondary response (Fig 6B). This suggests that 4-1BB-medaited increase of IL-2 is important for promoting the development of memory CD8^+^ T cells during the primary expansion of CD8^+^ T cells [27]. 4-1BB signaling itself may contribute to enhancing the primary expansion of CD8^+^ T cells and their survival. Feau et al. demonstrated that CD4^+^ T cells provide the help for memory formation of CD8^+^ T cells by activating DCs through CD40L-CD40 interactions rather paracrine production of IL-2 [22]. Since 4-1BBL^-/-^ mice show normal primary expansion of CD8^+^ T cells against influenza virus but decreased T cell numbers 3–5 weeks after the infection [28], it is possible that 4-1BBL on CD4^+^ T cells is also involved in providing the help for memory CD8^+^ T cell generation.

Bachman et al. reported that IL-2 during expansion phase of CD8^+^ T cells and antigen presence is detrimental for the T cells due to the increase of AICD, while IL-2 during the contraction phase promotes survival of CD8^+^ T cells [29]. One of the important functions of 4-1BB is protecting CD8^+^ T cells from AICD. Given that contradictory functions of IL-2 are due to the activation status of CD8^+^ T cells, 4-1BB signaling would strongly prevent the activated CD8^+^ T cells from AICD and thus, the increased IL-2 by 4-1BB triggering would be utilized only to enhance the expansion and differentiation of CD8^+^ T cells independent of their activation status.

Our novel findings in this study reveal that 4-1BB-mediated increase of IL-2 production from CD8^+^ T cells not only amplifies the effects of 4-1BB signaling in CD8^+^ T cells, but also appears to provide an environment in which 4-1BB-triggered CD8^+^ T cells can survive, proliferate, and differentiate independent of the help from CD4^+^ T cells. Collectively, our data demonstrate that 4-1BB triggering directly induces IL-2Rα and IL-2 expressions on CD8^+^ T cells via the PI3K/ERK pathway, and which activates the IL-2R-mediated signaling pathways in an autocrine manner. Therefore, 4-1BB triggering generates an environment in which the activated CD8^+^ T cells can preferentially proliferate and survive less dependent on the helper functions of CD4^+^ T cells. Our study may contribute to clarifying how 4-1BB signal modulates T cells *in vivo*, and thus addressing potential efficacy and safety issues in agonistic 4-1BB antibody-based cancer immunotherapeutics that we are actively pursuing.
